# Novel Tetracyclines Versus Alternative Antibiotics for Treating Acute Bacterial Infection: A Meta-Analysis of Randomized Controlled Trials

**DOI:** 10.3390/antibiotics8040233

**Published:** 2019-11-22

**Authors:** Shao-Huan Lan, Wei-Ting Lin, Shen-Peng Chang, Li-Chin Lu, Chih-Cheng Lai, Jui-Hsiang Wang, Chien-Ming Chao

**Affiliations:** 1 School of Pharmaceutical Sciences and Medical Technology, Putian University, Putian 351100, China; shawnlan0713@gmail.com; 2 Department of Orthopedic, Chi Mei Medical Center, Tainan 71004, Taiwan; aapriliaa@gmail.com; 3 Department of Physical Therapy, Shu Zen Junior College of Medicine and Management, Kaohsiung 82144, Taiwan; 4 Yijia Pharmacy, Tainan 70846, Taiwan; httremoon@ms.szmc.edu.tw; 5 School of Management, Putian University, Putian 351100, China; jane90467@gmail.com; 6 Department of Internal Medicine, Kaohsiung Veterans General Hospital, Tainan Branch, Tainan 71051, Taiwan; dtmed141@gmail.com; 7 Department of Internal Medicine, Division of Infection Disease, Kaohsiung Veterans General Hospital, Tainan Branch, Tainan 71051, Taiwan; hemletkimo@gmail.com; 8 Department of Intensive Care Medicine, Chi Mei Medical Center, Liouying 73657, Taiwan

**Keywords:** novel tetracycline, omadacycline, eravacycline, acute bacterial infection

## Abstract

This meta-analysis assessed the efficacy and safety of novel tetracyclines for treating acute bacterial infections. Data from PubMed, Web of Science, EBSCO, Cochrane databases, Ovid Medline, and Embase databases were accessed until 11 July 2019. Only randomized controlled trials (RCTs) comparing the efficacy of novel tetracyclines with that of other antibiotics for treating acute bacterial infections were included. Primary outcomes included the clinical response, microbiological response, and risk of adverse events (AEs). A total of eight RCTs were included, involving 2283 and 2197 patients who received novel tetracyclines and comparators, respectively. Overall, no significant difference was observed in the clinical response rate at test of cure between the experimental and control groups (for modified intent-to-treat [MITT] population, risk ratio [RR]: 1.02, 95% confidence interval [CI]: 0.99–1.05; for clinically evaluable [CE] population, RR: 1.02, 95% CI: 1.00–1.04; and for microbiological evaluable [ME] population, RR: 1.01, 95% CI: 0.99–1.04). No significant difference in the microbiological response at the end of treatment was observed between the experimental and control groups (for ME population, RR: 1.01, 95% CI: 0.99–1.03; for microbiological MITT population, RR: 1.01, 95% CI: 0.96–1.07). No difference was observed concerning the risk of treatment-emergent adverse events (TEAEs), serious adverse events, and discontinuation of treatment due to TEAEs and all-cause mortality between the two groups. In conclusion, clinical efficacy and safety profile for novel tetracyclines in the treatment of acute bacterial infections were found to be similar to those for other available antibiotics.

## 1. Introduction

Antibiotics are crucial for treating acute bacterial infections, and the prompt use of appropriate antibiotics can save the life of a patient with sepsis [1]. However, the emergence and dissemination of antibiotic resistance among commonly encountered bacteria in many types of infections, including pneumonia and intra-abdominal, urinary tract, and skin/skin structure infections, have drastically reduced the efficacy of most antimicrobial drugs [2,3,4,5,6,7]. Therefore, searching new antimicrobials to combat the threat of antibiotic-resistant bacteria is urgent.

Recently, two novel tetracyclines, omadacycline, (Nuzyra^®^, Paratek Pharmaceuticals, Boston, MA, USA) and eravacycline (Xerava^®^, Tetraphase Pharmaceuticals, Watertown, MA, USA), have been developed and approved by the Food and Drug Administration in 2018 [8]. Additionally, they are broad-spectrum antibiotics such as conventional tetracyclines that act against gram-positive, gram-negative, anaerobic, and atypical pathogens. Furthermore, they exhibit potent in vitro activity against multidrug-resistant organisms [9,10]. The clinical efficacy of omadacycline and eravacycline for treating acute bacterial infections is being evaluated in several randomized controlled trials (RCTs) since their development [11,12,13,14,15,16,17,18]. However, no consensus on the efficacy and safety of novel tetracyclines has been reached due to the lack of a systematic analysis and an updated meta-analysis. Therefore, we conducted this meta-analysis to provide a real-time evidence on the efficacy and safety of omadacycline and eravacycline for treating acute bacterial infections.

## 2. Methods

### 2.1. Study Search and Selection

All RCTs were identified through a systematic literature review of PubMed, Web of Science, EBSCO, Cochrane databases, Ovid Medline, and Embase until July 2019 by using the following search terms: “eravacycline”, “Xerava™”, “TP-434”, “omadacycline”, “Nuzyra”, “PTK-0796”, and “infection”. The inclusion criteria included (1) randomized controlled studies and (2) sturdy directly compared the clinical efficacy and safety of novel tetracyclines with those of other antimicrobial agents for treating adult patients with acute bacterial infections. Exclusion criteria included: (1) case reports, and abstracts presented at scientific conferences; (2) those including individuals younger than 18 years of age; (3) studies that only reported in vitro activity, animal studies, or pharmacokinetic–pharmacodynamic assessments; (4) case series without a control group; (5) trials that lacked randomized-control design. Two authors (S.P.C. and S.H.L.) searched and examined publications independently. A third author (C.C.L.) resolved any disagreement in time. The following data were extracted: year of publication, study design, type of infections, antimicrobial regimens, clinical and microbiological outcomes, and adverse effects. This systematic review and meta-analysis were conducted according to the preferred reporting items for systematic reviews and meta-analyses (PRISMA) statement.

### 2.2. Outcome Measurement

The primary outcomes of this meta-analysis included clinical response assessed at the test of cure (TOC) and end of treatment (EOT) visits, which was calculated as the portion of the patients with clinical response among analyzed populations. Clinical response was defined as the signs/symptoms of infection being sufficiently resolved and no further antibacterial therapy was required. Patients were categorized based on the occurrence of primary outcomes as follows: modified intent-to-treat (MITT), clinically evaluable (CE), and microbiologically evaluable (ME) populations. The intention-to-treat (ITT) population included all randomized patients, and the MITT population included all ITT patients who received any amount of the study drug. The CE population included all MITT patients who met the minimal disease definition of acute bacterial infections and had their clinical response assessed at the TOC visit. The ME population included all CE patients who had the baseline pathogen identified and microbiological response assessed. The microbiological MITT (mMITT) population included all MITT patients who met the minimal disease definition of clinical infection and had the baseline pathogen identified. The safety population included all patients who received any study therapy. Treatment-emergent adverse events (TEAEs) were defined as adverse events (AEs) that started during or after the first dose of the study drug administration or increased in severity or were associated with the study drug during the study.

### 2.3. Data Analysis

The Cochrane risk of bias assessment tool [19] was used to assess the quality of enrolled RCTs and the associated risk of bias. Review Manager, version 5.3, with the random-effects model was used for statistical analyses. The heterogeneity degree was assessed using the Q statistic generated from the χ^2^ test, and the heterogeneity proportion was assessed using the *I*^2^ measure. Heterogeneity was considered significant at *p* < 0.10 or *I*^2^ > 50%. Pooled risk ratios (RRs) and 95% confidence intervals (CIs) were calculated for outcome analyses.

## 3. Results

### 3.1. Study Selection

**Figure 1:**
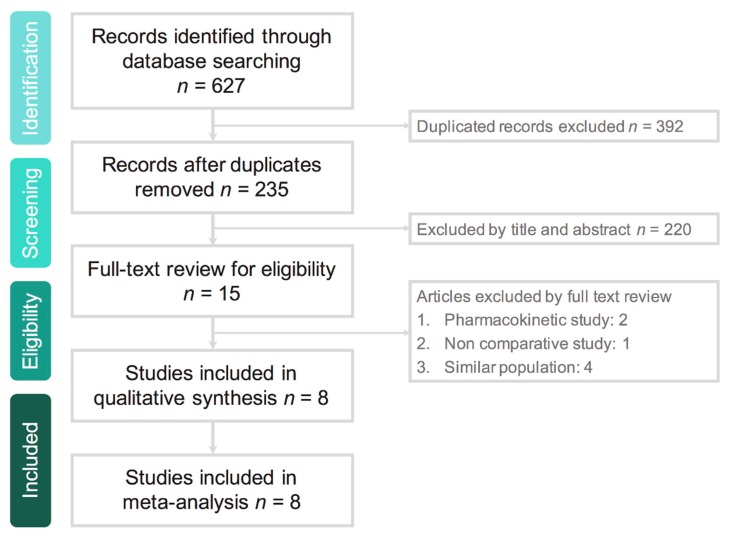
Algorithm for the screening and identification of studies.

Search results yielded a total of 627 studies from the following online databases: PubMed (n = 124), Web of Science (127), EBSCO (*n* = 43), Cochrane Library (*n* = 53), Ovid Medline (*n* = 128) and Embase (n = 163) (Appendix A). Overall, 392 duplicate studies were excluded. Additionally, 220 studies were found to be irrelevant after screening the title and abstract (based on the article type and language) and 15 studies after screening the full text. Eventually, eight RCTs [11,12,13,14,15,16,17,18] were selected for the meta-analysis (Figure 1).

### 3.2. Study Characteristics

**Figure 2:**
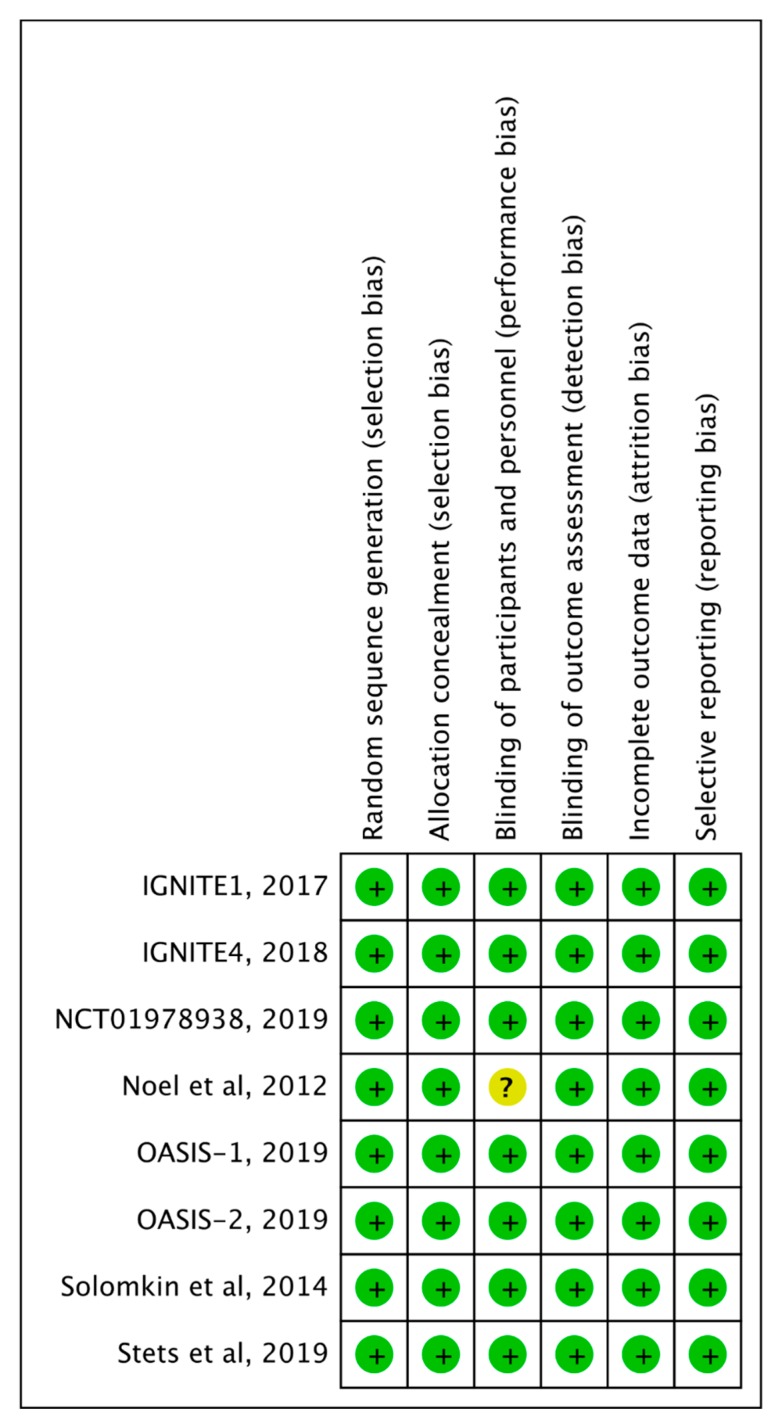
Risk of bias summary.

All eight included RCTs [11,12,13,14,15,16,17,18] were multicenter studies (Table 1). Each four studies used omadacycline [12,13,17,18] and eravacycline [11,14,15,16] as the study drug. Each three studies focused on acute bacterial skin and skin structure infections (ABSSSIs) [12,13,18] and complicated intra-abdominal infections (cIAIs) [14,15,16]. One study focused on complicated urinary tract infection (cUTI) [11], and the remaining study focused on community-acquired bacterial pneumonia (CABP) [17]. Overall, the experimental group comprised 2283 patients (omadacycline, *n* = 1195; eravacycline, *n* = 1088), and the control group comprised 2197 patients. Almost all risks of bias in each study were low (Figure 2).

### 3.3. Clinical Efficacy

**Figure 3:**
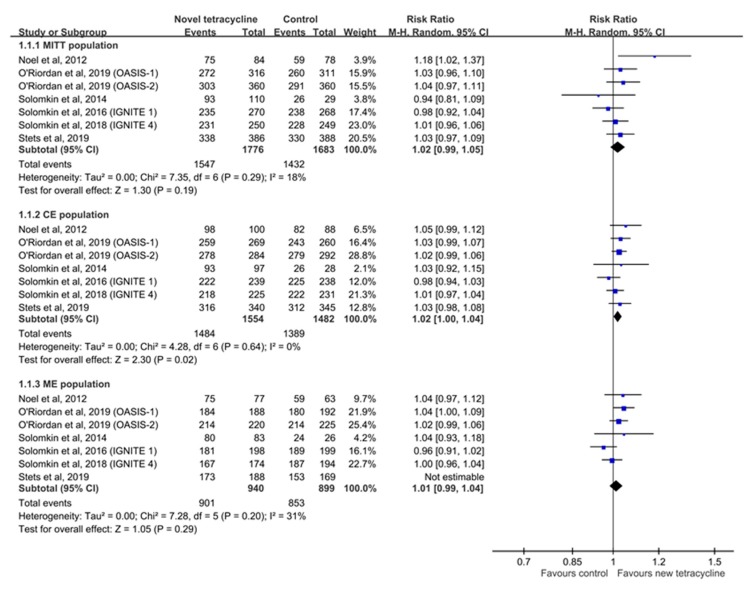
Forest plot of clinical response rate at the test of cure visit among modified intent-to-treat (MITT) population, clinically evaluable (CE) population, and microbiological evaluable (ME) population.

Overall, no significant difference was observed in the clinical response rate at TOC between the experimental and control groups (for MITT population, RR: 1.02, 95% CI: 0.99–1.05, *I^2^* = 18%; for CE population, RR: 1.02, 95% CI: 1.00–1.04, *I^2^* = 0%; and for ME population, RR: 1.01, 95% CI: 0.99–1.04, *I^2^* = 17%; Figure 3). In the mMITT population, similarity in the terms of the clinical response rate was observed between the experimental and control groups (RR: 1.00, 95% CI: 0.96–1.04, *I^2^* = 22%). In addition, the clinical response rate at EOT remained similar between the experimental and control groups (for MITT population, RR: 1.02, 95% CI: 0.99–1.05, *I^2^* = 18% and for CE population, RR: 1.02, 95% CI: 0.99–1.02, *I^2^* = 0%). Sensitivity analysis performed after deleting individual studies each time to determine the effect of a single dataset on the pooled RR revealed similar findings. In the subgroup analysis, the clinical response rate of omadacycline was non-inferior to that of comparators (for MITT population, RR: 1.04, 95% CI: 1.00–1.08, *I^2^* = 3%; for CE population, RR: 1.03, 95% CI: 1.01–1.05, *I^2^* = 0%; and for ME population, RR: 1.03, 95% CI: 1.01–1.06, *I^2^* = 0%), and the clinical efficacy of eravacycline was similar to that of comparators (for MITT population, RR: 0.99, 95% CI: 0.96–1.03, *I^2^* = 0%; for CE population, RR: 1.00, 95% CI: 0.97–1.03, *I^2^* = 0%; and for ME population, RR: 0.99, 95% CI: 0.96–1.02, *I^2^* = 0%).

Novel tetracyclines exhibited similar clinical efficacy to that of comparators for treating infections of both gram-positive (RR: 1.01, 95% CI: 0.97–1.05, *I^2^* = 0%) and gram-negative (RR: 0.99, 95% CI: 0.94–1.05, *I^2^* = 0%) aerobes. There was no exception for *Staphylococcus aureus* (RR: 1.03, 95% CI: 0.98–1.08, *I^2^* = 0%) as well as MRSA (RR: 1.04, 95% CI: 0.98–1.11, *I^2^* = 0%).

### 3.4. Microbiological Response

**Figure 4:**
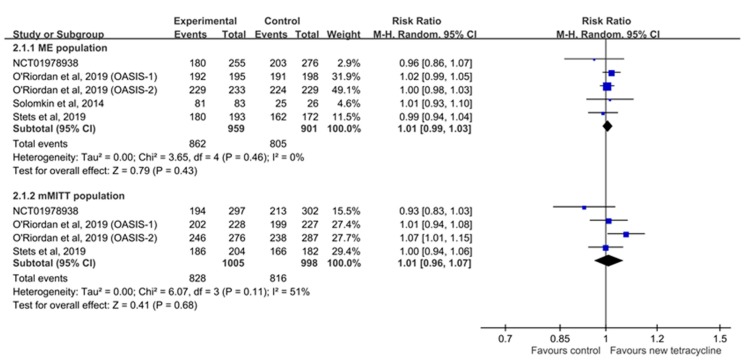
Forest plot of the microbiological response rate at the end of treatment visit between microbiologically evaluable (ME) and microbiologically modified intent-to-treat (mMITT) populations.

In the pooled analysis, no significant difference was observed in the microbiological response at EOT between the experimental and control groups (for ME population, RR: 1.01, 95% CI: 0.99–1.03, *I^2^* = 0% and for mMITT population, RR: 1.01, 95% CI: 0.96–1.07, *I^2^* = 51%; Figure 4). In addition, the microbiological response of novel tetracyclines at TOC was non-inferior to that of comparators (for ME population, RR: 1.03, 95% CI: 1.01–1.06, *I^2^* = 0% and for mMITT population, RR: 1.04, 95% CI: 1.00–1.09, *I^2^* = 0%). Furthermore, both omadacycline and eravacycline exhibited a microbiological response similar to that of comparators in subgroup analyses.

### 3.5. Risk of AEs

For common AEs, novel tetracyclines were associated with higher risks of nausea and vomiting than comparators (nausea, RR: 2.38, 95% CI: 1.16–4.89, *I^2^* = 87% and vomiting, RR: 2.13, 95% CI: 1.18–3.83, *I^2^* = 67%). Further subgroup analysis revealed that eravacycline was associated with higher risks of nausea and vomiting than the comparator (nausea, RR: 5.08, 95% CI: 1.96–13.11, *I^2^* = 55% and vomiting, RR: 2.33, 95% CI: 1.02–5.32, *I^2^* = 52%) but not omadacycline (nausea, RR: 1.40, 95% CI: 0.56–3.47, *I^2^* = 91% and vomiting, RR: 1.95, 95% CI: 0.77–4.96, *I^2^* = 80%).

**Figure 5:**
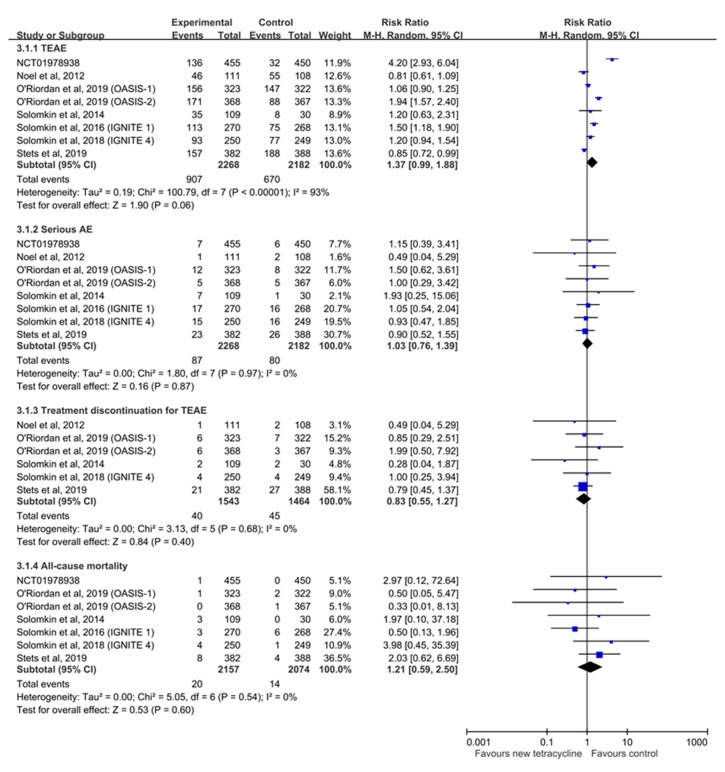
Forest plot of risks of treatment-emergent adverse events (TEAEs), serious adverse events, and discontinuation of treatment due to TEAE and all-cause mortality.

Overall, novel tetracyclines were associated with a similar risk of AEs as comparators (TEAE, RR: 1.37, 95% CI: 0.99–1.88, *I^2^* = 93%; serious AEs, RR: 1.03, 95% CI: 0.76–1.39, *I^2^* = 0%; and treatment discontinuation due to TEAE, RR: 083, 95% CI: 0.55–1.27; Figure 5). All-cause mortality did not differ between the experimental and control groups (RR: 1.21, 95% CI: 0.59–2.50, *I^2^* = 0%).

## 4. Discussion

Data from eight RCTs with 4480 patients were collated to compare the efficacy and safety of novel tetracyclines and other antibiotic regimens for treating acute bacterial infections, including cIAIs, ABSSSIs, CABP, and cUTIs. In the present study, we demonstrated that these novel tetracyclines could achieve a similar clinical response as other comparators, which is supported by the following evidence. First, the clinical response rate for novel tetracyclines, namely omadacycline and eravacycline, was similar to other comparative antibiotics. This similarity between novel tetracyclines and comparators was observed in various population analyses, MITT, CE, ME, and mMITT populations, and at different timings of assessment, TOC and EOT. Second, in subgroup analyses, both omadacycline and eravacycline exhibited non-inferior clinical efficacy than comparators. This finding is consistent with those of previous studies [20,21]. In the pooled analysis of OASIS-1 and OASIS-2, Abrahamian et al. [20] demonstrated that omadacycline was non-inferior to linezolid in early clinical response (86.2% vs. 83.9%; difference 2.3, 95% CI: 1.5–6.2) for treating ABSSSIs, and clinical responses were similar across different infection types—cellulitis or erysipelas and major abscess. Lan et al. [21] revealed that eravacycline had a clinical cure rate (88.7%, 559/630) similar to that of comparators (88.7% vs. 90.1%, RR: 0.99, 95% CI: 0.95–1.03) for treating cIAIs. Unlike these two reports [20,21], the present study included more RCTs and infection types, cUTI and CABP, to augment the knowledge regarding the usefulness of eravacycline and omadacycline. Third, the clinical efficacies of novel tetracyclines were similar to those of comparators across infections caused by different pathogens, even MRSA. A previous pooled analysis [20] of OASIS-1 and OASIS-2 revealed that omadacycline had similar efficacy to that of linezolid for treating infections caused by gram-positive anaerobes, including *S. aureus*, MRSA, *Streptococcus pyogenes*, and *S. anginosus*, gram-negative aerobes, and gram-negative anaerobes. In summary, all these findings indicate that novel tetracyclines, eravacycline, and omadacycline, can be as effective as other antibiotics for treating acute bacterial infections.

In addition to the clinical response, this meta-analysis demonstrated that the microbiological response rate for novel tetracyclines was comparable to that of comparators. This similarity in terms of the microbiological response between the experimental and control groups did not change with the timing of the outcome measure and study populations. These findings regarding the favorable microbiological response of novel tetracyclines have been supported by many in vitro studies [22,23,24,25,26,27,28,29]. Several global surveillance investigations [22,23,24,25] have revealed that omadacycline exhibited potent in vitro activity against gram-positive and gram-negative pathogens as well as was active against antibiotic-resistant organisms, such as MRSA, penicillin-resistant *S. pneumoniae*, and extended-spectrum β-lactamase (ESBL)-producing *Escherichia coli.* The potency of eravacycline was at least equivalent or 2- to 4-fold greater than that of tigecycline against Enterobacteriaceae, including ESBL-producing, carbapenem non-susceptible strains, and gram-positive cocci isolates [26,27,28,29]. Therefore, these findings regarding the microbiological response in this meta-analysis and previous in vitro studies can support the use of novel tetracyclines for acute bacterial infections.

Finally, the risk of AEs for novel tetracyclines was assessed. Nausea was the most common AE for novel tetracycline users, and novel tetracyclines were associated with a higher risk of nausea and vomiting compared with comparators. Further subgroup analysis revealed that high risks of nausea or vomiting were noted for eravacycline but not for omadacycline. However, compared with other antibiotics, novel tetracyclines had a similar risk of AEs in TEAEs, serious AEs, treatment discontinuation due to TEAEs, and all-cause mortality. All these findings indicated that gastrointestinal intolerance was the most common side effect of novel tetracyclines, especially eravacycline. However, novel tetracyclines were found to be as tolerable as other antibiotics.

This meta-analysis has some limitations. First, although we aimed to investigate the use of novel tetracyclines for treating all types of acute bacterial infections, we found only one study for cUTI as well as for CABP. Additional studies investigating the use of novel tetracyclines for various infection types are warranted. Second, we could not assess the association between in vitro activity and clinical response for each specific pathogen due to the unavailability of data. However, this deficit could be partially compensated by the results of several in vitro studies [22,23,24,25,26,27,28,29] that demonstrated the potent in vitro activity of novel tetracyclines.

## 5. Conclusions

In conclusion, clinical and microbiological responses for novel tetracyclines in the treatment of acute bacterial infections were similar to those for other available antibiotics. In the present analysis, eravacycline was associated with higher risks of gastrointestinal AEs, nausea, and vomiting, but overall, novel tetracyclines had a safety profile similar to that of other antibiotics. However, further research is warranted to investigate the role of novel tetracyclines in the treatment of antibiotic-resistant bacteria-associated infections.

## Figures and Tables

**Table 1:** Characteristics of included studies.

Study, Published Year	Study Design	Study Site	Study Period	Type of Infection	No of Patients		Dose Regimen	
					Study	Comparator	Study	Comparator
**Omadacycline**								
Noel et al., 2012	Randomized, controlled, evaluator-blinded study	11 sites in US	2007–2008	Complicated skin and skin structure infection	118	116	100 mg qd	Linezolid 600 mg q12h ± aztreonam
O’Riordan et al., 2019 (OASIS-1)	Double blind, randomized controlled trial	55 sites in US, Peru, South Africa and Europe	2015–2016	Acute bacterial skin and skin-structure infection	323	322	100 mg q12h x 2 doses than 100 mg qd	Linezolid 600 mg q12h
Stets et al., 2019	Double blind, randomized controlled trial	86 sites in Europe, North America, South America, the Middle East, Africa, and Asia	2015–2017	Community-acquired pneumonia in PSI risk II, III or IV	386	388	100 mg q12h x 2 doses than 100 mg qd	Moxifloxacin 400 mg
O’Riordan et al., 2019 (OASIS-2)	Double0blind, randomized controlled trial	33 sites in US	May 2017–June 2017	Acute bacterial skin and skin-structure infection	368	367	450 mg (oral) qd x 2 doses then 300 mg qd	Linezolid 600 mg (oral) q12h
**Eravacycline**								
Solomkin et al., 2014	Randomized, double-blind trial	19 sites in 6 countries	2011–2012	Complicated intra-abdominal infection	56 (1.5 mg/kg), 57 (1.0 mg/kg)	30	1.5 mg/kg or 1.0 mg/kg q24h	Ertapenem 1 g q24h
Solomkin et al., 2017	Randomized, double-blind trial	66 sites in 11 countries	2013–2014	Complicated intra-abdominal infection	270	271	1.0 mg/kg q12h	Ertapenem 1 g q24h
Solomkin et al., 2018	Randomized, double-blind trial	65 sites in 11 countries	2016–2017	Complicated intra-abdominal infection	250	250	1.0 mg/kg q12h	Meropenem 1 g q8h
NCT01978938	Randomized, double-blind, double-dummy, prospective study	99 sites in 18 countries	2014–2015	Complicated urinary tract infection	455	453	1.5 mg/kg q24h	Levofloxacin 750 mg q24h

PSI, Pneumonia severity index.

## Abbreviations

MITT, modified intent-to-treat; RR, risk ratio; CI, confidence interval; AE, adverse event; CE, clinical evaluable; ME, microbiological evaluable; RCT, randomized controlled trial; TEAE, treatment-emergent adverse event; TOC, test of cure; end of treatment, EOT; mMITT, microbiological modified intent-to-treat.

## Appendix A

**Table A1:** Search Strategy.

**PubMed search strategy—last searched on 11 July 2019**		**Results**
1	Search (((((Eravacycline[Title/Abstract]) OR Xerava[Title/Abstract]) OR TP-434[Title/Abstract]) OR Omadacycline[Title/Abstract]) OR Nuzyra[Title/Abstract]) OR PTK-0796[Title/Abstract]	172
2	Search Infection*[Title/Abstract]	1,343,862
3	Search (Infection*[Title/Abstract]) AND ((((((Eravacycline[Title/Abstract]) OR Xerava[Title/Abstract]) OR TP-434[Title/Abstract]) OR Omadacycline[Title/Abstract]) OR Nuzyra[Title/Abstract]) OR PTK-0796[Title/Abstract])	124
**Web of Science search strategy—last searched on 11 July 2019**		**Results**
1	Topic: (Omadacycline) OR Topic: (Nuzyra) OR Topic: (PTK-0796) OR Topic: (Eravacycline) OR Topic: (Xerava) OR Topic: (TP-434)	172
2	Topic: (Infection*)	1,382,483
3	#1 AND #2	127
**EBSCO search strategy—last searched on 11 July 2019**		**Results**
1	AB Eravacycline OR AB Xerava OR AB TP-434 OR AB Omadacycline OR AB Nuzyra OR AB PTK-0796	68
2	AB Infection *	489,510
3	S1 AND S2	43
**Cochrane Library search strategy—last searched on 11 July 2019**		**Results**
1	(Omadacycline):ti,ab,kw OR (PTK-0796):ti,ab,kw OR (Nuzyra):ti,ab,kw	33
2	(Eravacycline):ti,ab,kw OR (Xerava):ti,ab,kw OR (TP-434):ti,ab,kw	14
3	(Infection*):ti,ab,kw	108,094
4	(#1 OR #2) AND #3	42
**Ovid Medline search strategy—last searched on 11 July 2019**		**Results**
1	(Omadacycline or Nuzyra or PTK-0796 or Eravacycline or Xerava or TP-434).ab	174
2	Infection *.ab	1,468,481
3	1 and 2	128
**Embase search strategy—last searched on 11 July 2019**		**Results**
1	omadacycline:ti,ab,kw OR nuzyra:ti,ab,kw OR ‘ptk 0796’:ti,ab,kw OR eravacycline:ti,ab,kw OR xerava:ti,ab,kw OR ‘tp 434’:ti,ab,kw	217
2	infection*:ti,ab,kw	1,726,005
3	#1 AND #2	163
